# Exploring Potential Modifiers of the Association Between Neurodevelopmental Disorders and Risk of Bullying Exposure

**DOI:** 10.1001/jamapediatrics.2022.1755

**Published:** 2022-06-06

**Authors:** Evelina Käld, Linda Beckman, Valsamma Eapen, Ping-I Lin

**Affiliations:** 1 School of Population Health & Environmental Sciences, King’s College London, London, United Kingdom; 2 Department of Health Sciences, Karlstad University, Karlstad, Sweden; 3 School of Psychiatry, University of New South Wales, Sydney, Australia; 4 Mental Health Research Unit, South Western Sydney Local Health District, Liverpool, Australia

## Abstract

This survey study examines data from the National Survey of Children’s Health to determine which sociodemographic factors might modify the association between neurodevelopmental disorders and being bullied.

Children with a diagnosis of a neurodevelopmental disorder (NDD) are more likely to be bullied than their neurotypical peers. This exposure to bullying may predispose children to subsequent emotional^1^ and behavioral disturbances.^2^ Individual attributes that can modify the association between NDD diagnosis and bullying exposure may shed some light on targeted prevention programs. However, the roles of sociodemographic factors in bullying exposure remain inconsistent across different populations.^3^ Furthermore, little is known if these risk factors interact with the diagnosis of NDD to affect the risk of bullying exposure. The goal of this study is to understand which factors may modify the association between NDDs and the risk of being bullied.

## Methods

We extracted data for 71 800 children from the National Survey of Children’s Health 2016-2017,^4^ where NDD diagnoses were established based on parental reports. The study involved only deidentified data from the public domain and was exempt from ethics approval at Karlstad University. We compared the proportions of NDD contributions, including autism spectrum disorder (ASD) and attention-deficit/hyperactivity disorder (ADHD), Tourette syndrome, learning disability, intellectual disability, and epilepsy, to the risk of bullying exposure, to prioritize the NDDs associated with bullying exposure. Potential factors associated with the risk of bullying exposure were identified using logistic regression models that adjusted survey weights. Furthermore, we examined whether these factors could interact with the diagnosis of NDD to jointly influence the risk of bullying exposure using the following formula: odds of being bullied = β_0_ + β_1_ × X_a_ + β_2_ × X_b_ + β_3_ × X_a_ × X_b_, where X_a_ and X_b_ indicate the diagnosis of NDD and effect modifier (eg, age), respectively. β_3_ and its corresponding *P* value with .05 as the cutoff were used to evaluate the interaction effect. For the β_3_ coefficient, a positive value implies synergistic joint effects while a negative value indicates that the effect modifier attenuates the association between NDD and bullying exposure.

## Results

**Figure 1.**
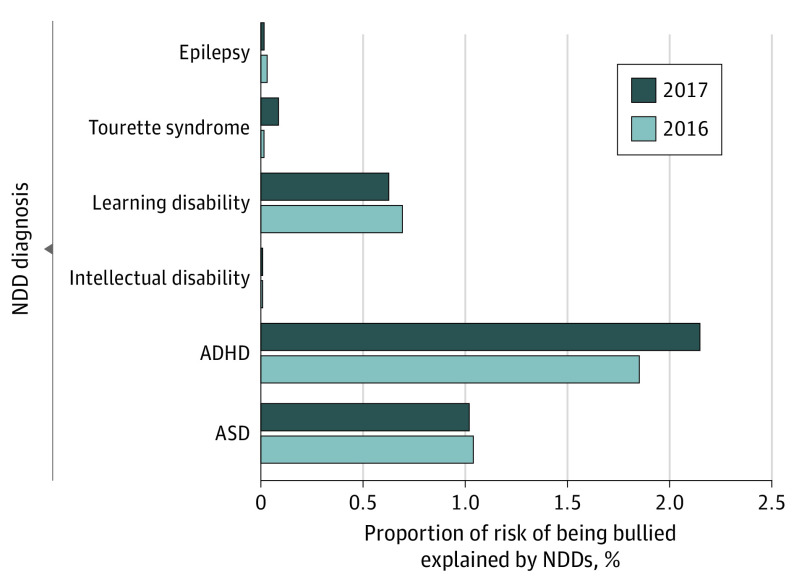
Relative Contributions of 6 Neurodevelopmental Disorders (NDDs) to Bullying-Related Experiences Squared values of partial correlations were used to estimate the percent proportion of contribution for each predictor to bullying exposure. The regression model adjusted for age, gender, parental education, and household income. ADHD indicates attention-deficit/hyperactivity disorder; ASD, autism spectrum disorder.

**Figure 2.**
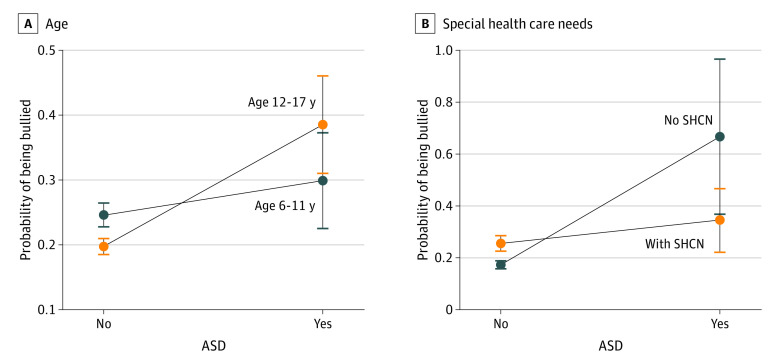
Association Between Bullying Exposure and Autism Spectrum Disorder (ASD) Modified by Age or Special Health Care Needs (SHCN)

The 2 NDDs that had the largest association with bullying exposure were ASD and ADHD (Figure 1). Special health care needs (SHCN) (OR, 1.9; 95% CI, 1.6-2.2), adverse childhood experience (OR, 1.6; 95% CI, 1.4-1.9), and disadvantaged neighborhoods (OR, 1.3; 95% CI, 1.1-1.5) were associated with a higher risk of being bullied, while older age was associated with a lower risk for only some children (OR, 0.8; 95% CI, 0.7-0.9). Notably, the interaction analyses showed that older children with ASD (age 12-17 years) were more likely to be bullied than younger children with ASD (6-11 years). In addition, disadvantaged neighborhoods and lack of SHCN further increased the association of ASD with the risk of bullying exposure. Figure 2 illustrates how age or SHCN interacted with the association between risk of being bullied and ASD. The association between bullying exposure and ADHD was not modified by any sociodemographic factors, neighborhood-related features, or SHCN.

## Discussion

Some individual attributes, such as age or SHCN, could exert different effects between children with ASD and their non-ASD peers. Individuals with ASD who do not have SHCN may be those with less severe symptoms. Compared with children with low-functioning ASD, they may be more likely to attend public schools, where bullying is more prevalent, especially in areas with low socioeconomic status.^5^ Note that the causal relationship between SHCN and bullying exposure cannot be clarified in our cross-sectional study. However, these findings are consistent with previous findings that children with high-functioning ASD who attended public schools are more likely to be bullied than other children with ASD.^6^ Older age could be a protective factor for children who do not have ASD, but it could be a risk factor for children with ASD. Additional research is indicated about bullying prevention programs targeting identified risk factors for children with ASD.

## References

[1] Bond L, Carlin JB, Thomas L, Rubin K, Patton G. Does bullying cause emotional problems? a prospective study of young teenagers. BMJ. 2001;323(7311):480-484. doi:10.1136/bmj.323.7311.480 11532838 PMC48131

[2] Matthews KA, Jennings JR, Lee L, Pardini DA. Bullying and being bullied in childhood are associated with different psychosocial risk factors for poor physical health in men. Psychol Sci. 2017;28(6):808-821. doi:10.1177/0956797617697700 28452573 PMC5461205

[3] Biswas T, Scott JG, Munir K, . Global variation in the prevalence of bullying victimisation amongst adolescents: role of peer and parental supports. EClinicalMedicine. 2020;20:100276. doi:10.1016/j.eclinm.2020.100276 32300737 PMC7152826

[4] Ghandour RM, Jones JR, Lebrun-Harris LA, . The design and implementation of the 2016 National Survey of Children’s Health. Matern Child Health J. 2018;22(8):1093-1102. doi:10.1007/s10995-018-2526-x 29744710 PMC6372340

[5] Juvonen J, Graham S, Schuster MA. Bullying among young adolescents: the strong, the weak, and the troubled. Pediatrics. 2003;112(6 pt 1):1231-1237. doi:10.1542/peds.112.6.1231 14654590

[6] Zablotsky B, Bradshaw CP, Anderson CM, Law P. Risk factors for bullying among children with autism spectrum disorders. Autism. 2014;18(4):419-427. doi:10.1177/1362361313477920 23901152

